# Cumulative effects assessment under the World Heritage and Ramsar regimes

**DOI:** 10.1007/s00267-025-02236-9

**Published:** 2025-08-14

**Authors:** Evan Hamman

**Affiliations:** Research Fellow, Centre for Environmental Governance, University of Canberra, Canberra, ACT Australia

**Keywords:** Cumulative Effects Assessment (CEA), International Environmental Law, Global Environmental Governance, World Heritage Convention, Ramsar Convention.

## Abstract

Managing cumulative pressures on socio-ecological systems presents as one of the foremost policy challenges of our time. Climate change, invasive species, development and pollution all have the potential to individually, and collectively, degrade the earth’s natural and cultural resources. International environmental law, a crucial element of global environmental governance, has a role to play in prompting and coordinating a response to cumulative effects. Hitherto, however, international approaches have been fragmented and unfocused, further complicating the already challenging application of Cumulative Effects Assessment (CEA) at the local level. This article examines how the two primary protected area treaties—the World Heritage Convention and the Ramsar Convention on Wetlands—have evolved to address the concepts of CEA and cumulative effects. It uncovers instances of ambiguity and fragmentation in treaty guidance material and concludes with a suggestion for further empirical research into how CEA could (or should) apply in the context of internationally protected areas.

## Introduction

Cumulative effects, or cumulative impacts, as they are sometimes known, refer to the incremental impacts of actions on the environment, when added to other past, present or reasonably foreseeable impacts (OECD 2006, UNESCO et al. (2022). More comprehensive definitions consider not just human impacts, but those of natural phenomena as well (Blakley 2021). Water basins, wetlands, forests, the marine environment, deserts and many other sensitive components of the Earth are interconnected in complex but increasingly well-documented ways. Activities such as extractive industry, agriculture, urban development, transportation and tourism all have the potential to negatively impact these systems, both individually and collectively. The resilience of many natural environments is further tested through the presence of invasive species and climate change, with the latter considered to be ‘the ultimate cumulative effect’ (EPA 2020). Combined, the accumulation of stresses has led to the mass loss of biodiversity (Newbold et al. 2015), depletion of water resources (Vörösmarty et al. 2010), degradation of the marine environment (Stockbridge et al. 2020) and impacts on local communities (Franks et al. 2010).

The scale at which threats materialise matters greatly, both in terms of time and space. The conservation of migratory species, for instance, is impacted by interventions along their migratory pathways (Webster et al. 2002; Ramsar 2010; CMS 2017). Moreover, through no fault of their own, small island states endure the incremental consequences of sea level rise despite greenhouse gas emissions released many miles away (Adelman 2016). The point is that accumulation is accumulation wherever and however it emerges. Yet, as this article reveals, and as others have pointed out (Nelson and Shirley (2023)), there is no comprehensive approach at the global scale to addressing cumulative effects. Rather, what has emerged is a patchwork of guidelines, policies, resolutions and decisions that reference a need to account for cumulative impacts, but only in particular contexts – biodiversity, heritage, wetlands and so on. Whist this likely reflects the fragmentation of international environmental law more generally (Morgan 2016), a lack of interoperability may undermine the application of cumulative effects assessment (CEA) on the ground, with environmental impact assessment (EIA) practitioners already struggling to apply concepts such as ‘impact’, ‘baseline’, ‘scale’, and ‘significance’ (Foley et al. 2017).

This article explores the extent to which international environmental law has evolved to embrace the notion of CEA, defined broadly as ‘the practice of systematically analysing cumulative environmental change’ (Smit and Spaling 1995; Gunn and Noble 2011). The article builds off recent work by Nelson and Shirley (2023) by examining the ‘extra-treaty material’ of two site-based global treaties: (1) the Convention Concerning the Protection of the World Cultural and Natural Heritage (the World Heritage Convention); and (2) the Ramsar Convention on Wetlands of International Importance (the Ramsar Convention). As Nelson and Shirley (2023) point out, CEA at the international level has been largely understudied, and therefore presents as an important research gap for further inquiry.

Studying the legal aspects of CEA at the international scale is important for a variety of reasons, not the least because it helps to build the foundations for ‘realizing the theoretical potential’ of CEA (Nelson, Shirley (2023), 167) as well as encouraging the use of consistent definitions and practices (Nelson and Shirley 2023, 165). Clear distinctions need to be made, for example, between terminology such as ‘direct’, ‘indirect’ and ‘cumulative’ impacts. Improving the language of international guidance material can help to facilitate this objective and address a major problem experienced in CEA of ‘insufficient clarity and guidance’ (Olagunju et al. 2021; Joseph et al. 2023). Sustained attention towards CEA at the global scale may also result in ‘norm diffusion’ (Busch and Jörgens 2013) including into other international frameworks that lack CEA provisions or policies (Nelson and Shirley 2023).

The article is structured in four parts. Part 1 introduces the notion of cumulative impacts under international environmental law. It explores the evolution of both EIA and cumulative impacts at the international scale highlighting the early work of the technical body to the Convention on Biological Diversity (the CBD)—the Subsidiary Body on Scientific, Technical and Technological Advice (SBSTTA). Part 2 of the article examines CEA under the Ramsar and World Heritage regimes. The methodological approach is discussed at the beginning of this Part including the decision to ground the analysis in the work of Gunn and Noble (2012); Dubé et al. (2013) and Blakley (2021) which focuses on assessment and management of impacts on so-called Valued Ecosystem Components (VECs). Part 3 of the article continues the discussion of this evaluation, comparing the two regimes and investigating the potential implications of their approach. Finally, Part 4 provides a brief conclusion.

## Part 1: Cumulative Effects and International Environmental Law

International environmental law can be conceived of as a collection of multilateral and bilateral legal instruments (treaties, agreements, protocols etc) that seek to govern one or more aspects of the environment.^1^ Over the last five decades, the evolution of environmental treaties, protocols and agreements has resulted in a veritable ‘maze’ of rules, practices and norms best described as ‘a complex topology’ (Kim 2013). The governance landscape is further complicated by the administrative practices of the different treaty regimes (e.g., resolutions, decisions, policy positions and so forth) some of which may be binding, and others which may not. Amongst the more well-known multilateral environmental agreements (MEAs) are the CBD, the Convention on Migratory Species (CMS) and the United Nations Framework Convention on Climate Change (UNFCCC). Treaties relating to the protection of heritage—such as the World Heritage Convention—cross over to focus on environmental issues through the inclusion of *natural heritage*. Similarly, MEAs like the Ramsar Convention have, in recent years, leant closer to recognising the cultural values of natural landscapes (Hamman 2025).

Most MEAs do not adequately address cumulative effects, at least on the surface. Indeed, the number of treaties or related instruments (guidelines, protocols, resolutions etc) specifically mentioning cumulative impacts or CEA is limited. A recent survey of MEAs concluded that only ten agreements (or corresponding protocols) contained specific provisions relating to CEA (Nelson and Shirley 2023). For the two MEAs studied in this article—the Ramsar Convention and the World Heritage Convention—there is no mention of cumulative impacts in the text of either treaty. Despite the lack of explicit wording (or perhaps because of it), some MEAs have evolved to recognise and respond to the challenge of cumulative impacts.

Beginning in 1990s, the concept of EIA, and more sophisticated assessment practices such as strategic assessment (or Strategic Environmental Assessment (SEA)), had started to become more commonplace in global policy discussions. Towards the end of the 1990s, a more sustained focus on cumulative effects was starting to appear in the decisions and resolutions of MEAs. This growth appeared to coincide with the mainstreaming of CEA theory and practice in domestic settings, most notably Canada (e.g. CEAA 1999), the United States (US) (CEQ 1997) and Europe (European Commission 1999). The focus appeared to be largely driven through the work of the technical bodies such as the SBSTTA under the CBD, and, to a lesser extent, the Scientific and Technical Review Panel (STRP) under the Ramsar Convention (see Part 2, below). In a 1995 recommendation on conservation of coastal and marine biodiversity, for instance, the SBSTTA suggested that States should take account of cumulative impacts when carrying out EIA for coastal and marine development (SBSTTA 1995). In 1998, their recommendation was carried forward into a formal decision of the Parties to the CBD where it was decided that States should deploy EIA to assess not only individual impacts, but cumulative effects as well (CBD 1998).

By 1999, further work by the SBSTTA stressed the importance of incorporating cumulative effects when assessing impacts of major projects on biodiversity (SBSTTA 1999). Shortly thereafter, voluntary guidelines were published by the CBD highlighting the need to clearly identify cumulative impacts on biodiversity throughout EIA processes (CBD 2002). By 2004, the CBD had well and truly embraced an ‘ecosystem-based’ approach (CBD 2004a; Waylen et al. 2014); a major component being that ecosystems were to be managed ‘within the limits of their functioning’ and that ‘cumulative effects of interventions over time and space should be assessed when considering [such] limits’ (CBD 2004a). The CBD’s approach towards EIA culminated in 2006, when the Parties to the Convention formally endorsed ‘voluntary guidelines’ for conducting EIA including several references to assessing cumulative effects through either (project level) EIA and/or (higher level) SEA (CBD 2006). Although voluntary in nature, the CBD’s Guidelines represented the most sophisticated international effort to date to ensure that cumulative effects on biodiversity were being considered through both EIA and SEA processes.

The work of the CBD was soon to be utilised by other treaties. For example, the 2002 Guidelines for incorporating biodiversity-related issues into EIA and SEA were formally adopted by Ramsar in 2002, and the 2006 voluntary guidelines were later adopted by the Bonn Convention on Migratory Species in 2017 (Ramsar 2002; CMS 2017, respectively). The World Heritage Convention, on the other hand, adopted its own unique approach to impact assessment; seemingly influenced both by the practice of EIA (through the involvement of nature conservation experts at the International Union for the Conservation of Nature (IUCN)) as well as Heritage Impact Assessment as result of cultural heritage advisors: the International Council on Monuments and Sites (ICOMOS); and the International Centre for the Study of the Preservation and Restoration of Cultural Property (ICCROM)).

The next section of the paper, Part 2, explores in greater depth how cumulative effects and CEA have been approached under both Ramsar and World Heritage.

## Part 2. CEA Under the Ramsar and World Heritage Conventions

### Methodological Approach

This research adopted a qualitative approach to the following question:

Has international environmental law sufficiently evolved to incorporate CEA in decision-making surrounding impacts on socio-ecological systems?

The question was developed following a desktop study of the available literature at the intersection cumulative effects, EIA and international environmental law. That review revealed a paucity of studies on whether international regimes had matured to address the question of cumulative effects, including, for example, whether CEA has been considered in treaty-related guidance or policy material. The approach builds off the recent work of Nelson, Shirley (2023) by exploring the ‘extra-treaty’ material of two site-based global treaties: the World Heritage and Ramsar conventions. The two regimes were selected as ‘cases’ for exploring the question (above) in greater depth. The World Heritage and Ramsar regimes, both established in the early days of international environmental law (1972 and 1971 respectively), are the only two site based global conservation treaties. With near universal acceptance, both are bounded in their focus (in the sense that they concern ‘protected areas’) and both adopt a listing approach to places of international significance (i.e., World Heritage and Ramsar sites). Both treaties cover some of the most sensitive and large-scale protected areas on the planet. These factors, combined, lend the regimes towards comparison on questions related to site-management, governance and EIA.

Having settled on the two regimes, the next step was to examine the text of each treaty. Keyword searches were conducted for phrases such as ‘cumulative’, ‘incremental’ ‘indirect’ and/or ‘additional’, in connection with ‘assessment’, ‘impacts’ and/or ‘effect’. In addition to examining the treaty text, other regime-related documents (hereinafter ‘regime material’) were examined using the same phrases. This approach reflected the idea that international agreements have evolved into dynamic settings where States progressively negotiate, reject and/or consent to new protocols, policies and procedures in addition to the text of the treaty itself (Brunnée 2002; Wiersema 2009). In the case of World Heritage, the additional regime material encompassed online decisions of the World Heritage Committee (including endorsed policies related to EIA and CEA) and the most recent Operational Guidelines (UNESCO 2023). The material was readily available online through UNESCO’s website (UNESCO World Heritage Centre 2025). In the case of the Ramsar Convention, regime material included COP Resolutions and Recommendations (including endorsed guidelines related to EIA and CEA) as well as reports from the STRP. As with World Heritage, all this material was available online (Ramsar 2025).

Relevant regime material was selected based on a keyword search of the terms listed above (e.g., cumulative, incremental, indirect etc). This material was then analysed using a content analysis approach. Content analysis is a research technique for making inferences from texts in the context of their use (Krippendorff (2018)). The inferences that arise can focus on the sender of the message, the message itself, or the audience (Weber 1990). This research was most interested in the message itself. The analysis adopted an ‘interpretative approach’ that allowed for both manifest and latent content to be considered (Drisko and Maschi 2016). For the identified material, it was necessary to adopt an analytical framework by which the ‘sufficient evolution’ of CEA could be evaluated. Although a variety of ‘best practice’ CEA approaches are available for this purpose, this research settled on the ‘four-phase approach’ towards assessment of impacts on VECs set out in Gunn and Noble (2012). Whilst there is no universally accepted approach towards CEA (Smit and Spaling 1995; Parr 1999; Xue, Hong and Charles 2004), Gunn and Noble have logically arranged the basic steps for CEA in a way that is broadly consistent with other approaches (e.g., Clark 1994; Dubé et al. 2013; Jones 2016).

The working thesis was that in a mature EIA (CEA) system, one might expect to see each of the four stages adequately addressed - either explicitly or implicitly - within the content of the regime material. As to what was ‘adequate’ or ‘sufficient’, guiding questions were developed drawing on criteria for good practice in CEA set out in Blakley (2021); Gunn and Noble (2012); Noble (2010); and Beanlands and Duinker (1983). These questions were used as guides only and not strictly applied in the content analysis. For example, if a document contained evidence of an intention to establish a scoping phase, though utilising other terminology, this was deemed acceptable for the purposes of the conclusion. The intention determination was guided by the ‘Rationales’ in Column 3 of Table 1.

**Table 1:** Evaluative Framework for examining the Regime Material under the World Heritage and Ramsar Conventions. Adapted from Gunn and Noble (2012); Dubé et al. (2013) and Blakley (2021)

CEA Stage	Description	Rationale	Search Terms	Guiding Questions
*Scoping*	Scoping the project to identify the incremental direct and indirect effects of the project on VECs.	To identify cumulative effects of projects on components of the receiving environment including all that will be included/excluded when evaluating impact.	Scoping, early evaluation, terms of reference, foreseeable, future, temporal and spatial etc.,	• Is the scoping approach distinct from project-based EIA?
				• Does it include both biophysical and sociocultural components?
				• Does it consider both human-induced and natural disturbances as within scope?
				• Does the scoping approach seek to capture all reasonably foreseeable future projects?
*Retrospective Analysis*	Assessing historical VEC conditions and analysing trends and changes in conditions over time and against thresholds.	To establish a baseline from which to evaluate cumulative effects, including how VEC conditions have changed over time.	Baseline, historical, past, condition, retrospective, allowable limits of change, thresholds, management targets, etc.,	• Is the analytical approach able to identify past and present cumulative effects?
				• Does it seek to establish a baseline and characterise trends and changes over time?
				• Does it encourage the identification of thresholds/targets against which cumulative change can be assessed?
*Prospective Analysis*	Connecting the project and other actions in the region to the VECs in terms of potential condition changes and predicting how they may respond to additional stress.	To predict how VECs respond to additional stress or disturbance.	Prospective, modelling, prediction, additional stress or disturbance, summing up, scenario analysis, future exploration etc.,	• Is the time scale of analysis able to capture impacts over the project’s life?
				• Are the techniques used capable of capturing the complexities and uncertainties of future developments?
				• Has the baseline analysis been used to inform predictions about cumulative impacts?
				• Is the analysis centred on the total effects on VECs and the project’s regional environment?
*Significance Determination*	Evaluating the significance of cumulative effects on VECs.	To determine how much more change in VEC conditions is tolerable, especially in the context of ecological limits or management targets.	Evaluation, tolerable change, significance, relative magnitude, etc.,	• Is the significance of a project’s cumulative effects measured against a past reference condition and not simply the current disturbed condition?
				• Are the cumulative effects adequately described and justified (e.g. through regulatory thresholds, environmental policies, expert evaluation, public concerns, etc.)?
				• Has a final determination been made about whether cumulative effects are acceptable/tolerable?

### The Evolution of CEA under the Ramsar Convention

The Ramsar Convention on Wetlands (Ramsar) was agreed to in the city of Ramsar, Iran in 1971. Its original focus, as evidenced through its original title, was the protection of wetlands as habitat for migratory waterbirds. However, over the last two decades, Ramsar has evolved to focus on human aspects of wetland systems (Bridgewater and Kim 2021); including the cultural values they exhibit (Hamman 2025).

Whilst the text of Ramsar does not refer to cumulative effects, several initiatives since the 1990s have highlighted the importance of addressing cumulative (or at least ‘indirect’) impacts. Much of this work has taken place specifically under the evolution of the ‘wise use’ concept. For example, at the fourth meeting of the Parties in 1990 (COP4) in Montreux, Switzerland, guidelines were adopted for the implementation of wise use (Ramsar 1990). Amongst other things, those guidelines recommended Parties assess all ‘projects upstream of [a] wetland, those in the wetland itself, and other projects which may affect the wetland’ (Ramsar 1990). At the same time, a technical working group was established to further explore the key concept of wise use, and, in Kushiro, Japan (COP5), the Parties formally resolved to implement wise use in a more systematic and effective manner (Ramsar (1996a)).

The efforts of the wise use working group and other technical experts were on full display in 1996 in Brisbane, Australia. At COP6, the Contracting Parties referenced the work done on EIA under Ramsar and highlighted that impact assessment should ‘not be restricted to individual projects’ but ‘should also address the cumulative effects of several projects’ impacting wetlands (Ramsar 1996). Increased advocacy on EIA came from prominent wetland experts (see e.g., Pritchard 1996 and Bagri and Vorhies 1999) who continued to advance the idea of utilising broader impact assessment techniques and making them specific to wetlands. The Ramsar Convention was not starting afresh in this regard, as there was already a vast amount of EIA literature and practice from which to draw upon (Pritchard 1996).

By the turn of the century, Ramsar had begun developing stronger ties with EIA practitioners, as evidenced through the June 2001 Memorandum of Understanding (MOU) with the International Association for Impact Assessment (IAIA) (Ramsar 2010, 8). A pivotal moment came in 2002, when the CBD’s Guidelines for incorporating biodiversity-related issues into EIA were formally adopted by the Parties to Ramsar (Ramsar 2002). The CBD’s approach was said to be ‘fully appropriate for application’ in the Ramsar context (Ramsar 2002, 5).

In 2006 the CBD adopted further guidance related to EIA (CBD 2006). The updated guidance was subsequently adopted by Ramsar in 2008 (Ramsar 2008). As was the case with the 2002 Guidance, Ramsar’s technical body—the STRP—provided annotations to ensure the uniqueness of wetland systems would be considered. The updated guidelines contained a more poignant focus on cumulative impacts and also attempted interlinkages between cultural, social and environmental impact assessment, something considered lacking in the first edition of the CBD’s Guidelines (Ramsar 2010, 6). The CBD’s updated 2006 guidelines, as endorsed and annotated by Ramsar in 2008, subsequently formed the basis of the Convention’s current approach to EIA (Ramsar 2010).

### Evaluating Ramsar’s Approach To CEA

In relation to stage one (an adequate scoping process), the current Ramsar guidance does include a comprehensive discussion on the need for scoping (after ‘screening’, Ramsar 2010, 14). The purpose is stated to identify the potential impacts on wetlands that are relevant to then conduct further assessment (Ramsar 2010, 14). Cumulative effects are specifically mentioned in this scoping phase, including highlighting the need to determine the spatial and temporal scales of influence regarding biophysical change on wetlands (Ramsar 2010, 24). The guidance further suggests a ‘broad scale’ is often needed, noting that the appropriate context at which to think about wetland impacts should usually be that of the watershed or river catchment (Ramsar 2010, 24).

Whilst Ramsar’s guidance mentions cumulative effects at the scoping stage, no comprehensive approach to CEA is outlined. Indeed, no clear distinction is made between related terms such as ‘effects’, ‘impacts’, ‘threats’ or ‘indirect impacts’ (Ramsar 2010, 26, 28, 31) - although it does distinguish between direct and indirect ‘drivers of change’ (Ramsar 2010, 50). Important language is used interchangeably, and, unlike the World Heritage Convention, below, there is no attempt at defining cumulative effects. In addition, it seems that less emphasis is placed on identifying cumulative effects on socio-cultural values (at least when contrasted with those of the biophysical). The inclusion of both biophysical and socio-cultural components is fundamental to the scoping stage (Gunn and Noble 2012) and it is increasingly recognised that local communities should be central to the identification of ‘valued components’ in impact assessment (Kwon, Rutherford and Gunton 2024). Whilst there is reference to the need to assess impacts on ‘ecosystem services of social value’ (Ramsar 2010, 16) and to the participation of Indigenous communities (by cross reference to the CBD’s *Akwé: Kon Voluntary Guidelines*) that assessment takes place within the broader context of ‘biodiversity’ without clear guidance as to unravelling the complex links between public participation, the selection of valued components and cumulative effects on society and culture at the scoping stage. The prioritisation of the biophysical is perhaps unsurprising, given Ramsar’s adoption of the CBD’s approach – bounded as it is within the aim of reversing biodiversity loss and the confusion is compounded by the cross-reference to the *Akwé Kon Guidelines* as opposed to a bespoke Ramsar-specific approach to EIA.

In relation to the requirement for a retrospective analysis, Ramsar’s guidance does go a long way to achieving this. The guidance highlights the importance of defining baseline conditions (Ramsar 2010, 24), including the need for an analysis of past and likely future conditions of the wetland in question. Cross reference is made to the CBD’s *Akwé: Kon Voluntary Guidelines* (Ramsar 2010, 12, 27) which also highlights the need for baseline studies in consultation with affected Indigenous and local communities (CBD 2004b, 16). Combined, these measures form a basis for identifying baseline conditions of the biophysical environment, and to a lesser extent, those of the socio-cultural context.

With regard to the need for a prospective analysis, Ramsar’s guidance does highlight the need to predict the response of biophysical values (again, seemingly prioritised over socio-cultural) to cumulative pressures (Ramsar 2010, 14). For example, the guidance refers to the need to predict effects of ‘direct drivers’ of change on wetland ecosystems, as well as to consider prospective changes within the broader temporal and spatial context of a wetland (e.g., at the watershed or river basin scale).

Finally, in relation to the requirement for a significance determination, the guidance notes the need for a final significance judgment in order to establish how much change is tolerable (Ramsar 2010, 28, 46). It further highlights the evaluation of the significance of impacts, including determining whether impacts are acceptable to wetland stakeholders (Ramsar 2010, 28).

### Evolution of CEA under The World Heritage Convention

The 1972 World Heritage Convention is the only multilateral agreement that concerns both cultural and natural heritage. Broadly speaking, States Parties under the Convention nominate sites for inclusion on a List of World Heritage (the WHL or ‘the List’). Inclusion is based upon the extent to which properties evince Outstanding Universal Value (OUV) which subsequently becomes codified in ‘Statement of OUV’.^2^ OUV is not defined in the text of the treaty, but paragraph 49 of the Operational Guidelines (UNESCO 2023) defines OUV to mean:

cultural and/or natural significance which is so exceptional as to transcend national boundaries and to be of common importance for present and future generations of all humanity.

As with Ramsar, the text of the World Heritage Convention does not mention impact assessment, nor for that matter, cumulative impacts or CEA. Over the last five decades, however, and particularly since the early 2000s, the decisions and guidance material of the Convention have evolved to embrace both EIA and Heritage Impact Assessment (HIA). A major component of both EIA and HIA has now become the consideration of cumulative impacts affecting heritage (UNESCO et al. 2022). The co-evolution of EIA, HIA and CEA has occurred alongside the (increasingly) detailed material in the Operational Guidelines (UNESCO 2023) as well as additional material produced by the scientific experts within the regime’s institutional structure (see e.g., ICOMOS 2011; IUCN 2013; UNESCO et al. 2022). The current (2022) guidance on EIA and HIA is discussed in more detail below, but a few preliminary points on the evolution of EIA and HIA under the regime are worth making.

The early days of the World Heritage Convention were focused largely on identifying and listing properties which showcased OUV, including negotiating the challenging question of which sites attracted ‘universal significance’ (i.e. the ‘U’ in OUV). The first meaningful attempt at engaging with cumulative impacts came, not in the form of impact assessment, but in the ‘recommendation’ that when nominating sites for inclusion on the WHL, a ‘buffer zone’ should be established (UNESCO 1978, 12). At the time, buffer zones were (and still are) a key strategy for protected area management, especially in the United States (e.g., Leopold et al. 1963) as well as through UNESCO’s Man and Biosphere program (Ebregt and Greve 2000). The idea of establishing a buffer zone *before* a site was listed became an integral part of the WHL nomination process (e.g., UNESCO 1983; UNESCO 1984) and continued that way throughout the 1990s and early 2000s.

Since about the late 1980s, a noticeable shift occurred in the decisions of the World Heritage Committee—the key decision-making body under the Convention—as well as the work of its Advisory Bodies—IUCN, ICCROM and ICOMOS. That shift entailed a move towards recognising and responding to threats faced by properties already on the List. Numerous categories of threats facing World Heritage properties have been reported since the 1990s, including: weak management and institutional regimes; building and development pressures, social and cultural (mis)uses of heritage, threats from transportation infrastructure, overuse of biological resources, physical resource extraction (such as oil and gas), increasing pollution, and the impacts of climate change and invasive species (Brown et al. 2019).

**Fig. 1:**
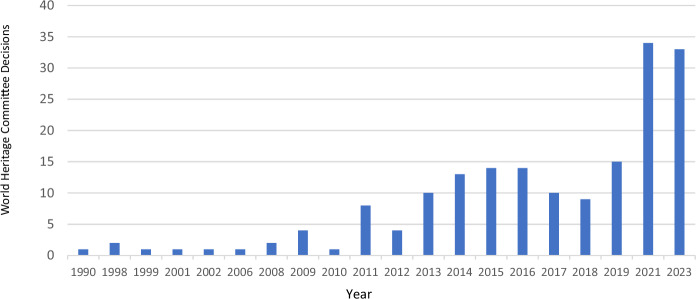
Number of decisions of the World Heritage Committee recognising World Heritage sites facing cumulative impacts between the years 1990 and 2023. Source UNESCO 2025

During the same period, a steady rise was observed in the total number of sites being included on the List of World Heritage in Danger (Brown et al. 2019; Hølleland et al. 2019), and by the early 2000s, deletion from the WHL had occurred for the first time. As a response to the growing list of threats, UNESCO and the Advisory Bodies raised the need to better predict and protect against cumulative impacts, specifically. From about 2000 onwards, the World Heritage Committee established a pattern of taking formal ‘note’ of cumulative effects facing at-risk properties (Fig. 1), especially in the context of large scale socio-ecological systems such as: Wood Buffalo National Park (Canada); the Great Barrier Reef (Australia); the Tropical Rainforest Heritage of Sumatra (Indonesia); Doñana National Park (Spain); Lake Turkana National Parks (Kenya); Manas Wildlife Sanctuary (India); Three Parallel Rivers of Yunnan Protected Area (China); and Lake Baikal (Russia).

In recognition of the increased number and complexity of sites facing cumulative impacts, UNESCO and the Advisory Bodies began developing more out-ward facing policy material. In 2011, for example, ICOMOS, the advisory body for cultural sites under the Convention, released guidance on impact assessment for cultural World Heritage sites (ICOMOS 2011). The guidance mentioned the need for HIA to consider both indirect and cumulative effects on culture as part of a ‘defendable system for assessing/evaluating impacts.’ However, it stopped short of providing a clear definition of cumulative (and indirect) impacts and did not articulate a comprehensive approach for conducting CEA.

Likewise, in 2013, the IUCN—the natural heritage advisor under the Convention—developed its own (separate) guidance on conducting EIA for proposals affecting natural World Heritage sites (IUCN 2013). As with ICOMOS’ guidance, cumulative impacts were mentioned as a component of assessing the likely effects of development, but no comprehensive approach was put forward for what CEA was, nor how it should be conducted. By the same token, no clear distinction was made between direct, indirect and cumulative impacts.

In 2015, in Bonn, Germany, the Committee formally highlighted the benefits of improved HIA and EIA acknowledging the prevalence of cumulative impacts affecting many World Heritage sites (UNESCO 2015). A three-year project between the Advisory Bodies and UNESCO was funded to explore improvements towards impact assessment. In 2022, an integrated guidance document for conducting impact assessment in a World Heritage context was released (UNESCO et al. 2022). The guidance merged and updated the two previous documents produced by IUCN and ICOMOS and remains the Convention’s current approach.

### Evaluating World Heritage’s Approach To CEA

In relation to the first element of CEA, involving an adequate scoping process covering both biophysical and socio-cultural factors, it seems clear that the 2022 guidance does provide a relatively comprehensive approach. It notes, for instance, that scoping should be used to identify ‘wider changes’ affecting OUV and include them in impact assessment in order to account for ‘all direct, indirect and cumulative impacts’ (UNESCO et al. (2022), 22). Further, the guidance suggests that scoping is to include both temporal and geographical considerations surrounding both natural and cultural heritage, including threats to the World Heritage property and its buffer zone.

In relation to the second stage of an adequate baseline assessment, it seems clear the approach of World Heritage stresses the importance of establishing a baseline, including describing the past, present, and likely future conditions of the property. In particular, the guidance highlights that consideration of potential future changes and threats, including other planned projects and regional trends near, are ‘essential for identifying cumulative impacts’, including where the effects may be more significant when combined with other past, present, and future actions (UNESCO et al. 2022, 37).

As regards the third stage, the guidance provides relatively clear coverage of the need to conduct a prospective analysis, including predicting how OUV is likely to respond to cumulative pressures. Further, the guidance notes that the identification and prediction of impacts is at the heart of impact assessment (UNESCO et al. (2022, 40) and reminds States that when identifying impacts:

it is important to remain aware of how a World Heritage property is interconnected with its buffer zone and wider setting, and that it cannot be viewed in isolation. All direct, indirect and cumulative impacts occurring in the entire area of influence of the proposed action need to be identified and assessed for their potential to impact the World Heritage property (UNESCO et al. (2022, 41).

Finally, it is clear that the guidance provides a structured approach for evaluating the significance of impacts on World Heritage, recommending that States categorise them as neutral, minor, moderate, or major (UNESCO et al. (2022),84). It further includes steps for defining the change that will occur to identified attributes, including considering factors such as reversibility, longevity, and the quality of change (UNESCO et al., 86).

## Part 3. Discussion

On the face of it, both Ramsar World Heritage have adopted approaches towards cumulative effects that broadly align with the four steps used in the methodological approach (above). Accordingly, it may be concluded that both regimes have evolved to at least attempt to proceduralise, through ‘approved’ guidance material, the concept of CEA within the broader context of EIA. However, there are additional points that are worth making with respect to how well the regimes articulate and seek to apply their respective positions. The below may be considered areas for ‘nudging’ the regime material in a more comprehensive direction in light of the available literature on CEA.

### Overcoming Ambiguity in Guidance Material

Ambiguity has long been a concern for EIA practitioners, not just in guidance material (Jones 2016) but in regulation and legislation as well (Arabadjieva 2017; Roudgarmi 2018). From the discussion above, although it seems clear that both the Ramsar and World Heritage regimes have engaged, and continue to engage, with the challenge of cumulative effects, there remain a few key opportunities where ambiguity could be addressed.

First, the approach of the World Heritage Convention, when compared with Ramsar, seems to be better articulated and defined for the context in which it is intended; that is, assessing and responding to direct, indirect and cumulative impacts on OUV. The World Heritage guidance clearly places OUV at its core and elevates the question of cumulative impacts to one of its ‘principles’ (UNESCO et al. (2022, 9). Moreover, the World Heritage material goes further to explain the difference between CEA in the context of SEA (as focusing on whether thresholds or standards are exceeded) and that of CEA in the context of (project-based) EIA/HIA (that is, to assess impacts of other projects jointly with project impact) (UNESCO et al. 2022, 21). It also (helpfully) defines the term ‘cumulative impact’ separating it from the terms ‘direct impact’ and ‘indirect impact’ (Table 2).^3^

**Table 2:** Relevant definitions ‘impacts’ under the 2022 World Heritage Impact Assessment Guidance

Indirect Impact	Indirect impacts are impacts on the environment which are not a direct result of the project, often produced away from or as a result of a complex pathway. Sometimes referred to as ‘second’ or ‘third-level’ impacts, or ‘secondary’ impacts.
Direct Impact	A direct impact is the result of a cause-and-effect relationship between a project and a specific attribute of World Heritage or other environmental components.
Cumulative Impact	A cumulative impact results from the environmental impacts of a project combining with the same environmental impacts of other past, existing or reasonably foreseeable future projects or activities, including those that may be enabled by the project.

In contrast, Ramsar’s approach does not distinguish between indirect or cumulative impacts. Rather, the terms are used loosely, and in an aggregate way (i.e., cumulative *and* indirect impacts), implying they are somehow related yet still distinct. In addition, the key words of ‘threat’, ‘impact’ and ‘effect’ are used interchangeably throughout the Ramsar material, complicating the already complex vernacular employed by both CEA and EIA across the world. A lack of basic definitions can cause problems in practice. For example, as Gunn and Noble (2011) report, one of the most often identified challenges in conducting CEA is that there is no basic agreement on what constitutes cumulative effects in the first place.

A second point to note is that unlike the World Heritage Convention, the Ramsar guidance does not clearly describe how CEA is to be differentiated within the context of (project based) EIA and (higher level) SEA. Whilst there are those that advocate that CEA is best undertaken regionally, rather than project-by-project (Jones 2016,) others suggest that CEA should be an integral part of both the project and strategic scale (Therivel and Ross 2007). Notwithstanding debates about when and how CEA should be utilised, it seems clear there are differences in applying CEA at different scales (whether project, regional or strategic). Ambiguity is likely to complicate (or over-simplify) matters for practitioners given approaches towards project-specific CEA are often not be readily transferable to other scales. As Hegmann (1999), 62) reports, in the context of regional assessment:

Regional [CEA] allows its practitioners to run in an open space of unfettered (albeit still tied to some budget) explorative inquiry, a space closed to project assessments. Unfortunately, many of the expectations heaped on project [CEA] are based on concepts that may thrive in a regional [CEA]…although there is on occasion common ground, the project and regional versions of [CEA] are far too different.

By the same token, a lack of clarity with respect to the use of CEA at the strategic scale also creates challenges. As Gunn and Noble (2011) have found, even though SEA may seem more aligned with the thinking around cumulative impacts and CEA, the two notions ‘do not [necessarily] blend seamlessly or effortlessly’. In the end, addressing ambiguity seems necessary, particularly in the Ramsar context, regarding how cumulative impacts are defined, and how and when CEA should be integrated into the different scales at which EIA is conducted.

### Valuing the Biophysical and the Socio-Cultural

One of the more challenging aspects of environmental management, not just in the case of impact assessment, but more broadly, is coming to terms with the interrelationship between cultural and natural values within a landscape. Whilst practitioners and governments have increasingly recognised the connection between culture and nature, effective frameworks for holistically marrying the two continue to evade practitioners and decision-makers (see e.g., Azzopardi et al. 2023). That is not to suggest that managing impacts on natural attributes, in and of itself, is straightforward (Therivel et al. 2021). But rather, socio-cultural values are likely to be less readily identifiable, more politically entangled and subject to data deficiencies.

Currently, within the framework of the Ramsar Convention, the focus on identifying cumulative effects appears weighted towards biodiversity, without sufficient attention directed to the impacts on cultural, recreational or spiritual components. Whilst there is mention under Ramsar of the need to engage with Indigenous stakeholders through cross reference to the CBD’s *Akwé: Kon Voluntary Guidelines* identifying and managing impacts on biodiversity remain the dominant focus. A lack of a more explicit approach towards socio-cultural values compounds the unfortunate reality that CEA tends to overlook Indigenous values, by either omitting them, overgeneralising them, or defining them inappropriately (Muir 2022; Kwon, Rutherford and Gunton 2024).

The issue of assessing and mitigating impacts on culture, more generally, has become a critical one for the Ramsar regime. A noticeable shift over the last three decades has occurred under Ramsar towards the more ‘human-centric’ benefits of wetlands and away from the pure conservation of biodiversity (Bridgewater and Kim 2021; Hamman 2025). Yet, whilst the guidance material for describing a wetland’s ecological character has adequately evolved (Hamman 2025), the material with respect to EIA appears outdated and deficient with respect to the question of identifying and managing cumulative impacts on social and cultural values of wetland environments. The World Heritage Convention, on the other hand, seems to adopt a more nuanced and defendable approach. It is likely that World Heritage’s approach has benefited from regime’s unique (long-held) focus on both natural and cultural heritage and the interrelationship between EIA and HIA.

That said, it is important to realise that both EIA and HIA remain largely distinct processes in practice. Recent attempts have been made to better integrate cultural heritage into environmental evaluation (Azzopardi et al. 2023), but the challenges of integrating the two, including with respect to CEA are considerable. As ICOMOS reported in 2011:

EIA frequently disaggregates all the possible cultural heritage attributes and assesses impact on them separately, through discrete receptors such as protected buildings, archaeological sites, and specified view-points with their view cones, without applying the lens of OUV to the overall ensemble of attributes (ICOMOS 2011).

More recently, as Strecker (2024) has highlighted, there are significant issues in the conventional practices of EIA and HIA that still tend to emphasise the physical environmental elements of a landscape prioritising the biophysical and the material. In the context of heritage, this includes bias towards the disciplines of archaeology and architecture, while often overlooking intangible cultural heritage (Strecker 2024). Strecker (2024, 253) writes:

Cultural heritage is often narrowly construed in the assessment process as discrete, physical heritage, something that can be enumerated, measured and mitigated against via excavation or removal, separate from social and cultural impact.

### Negotiating the Complexities of Scale

The guidance material from both Ramsar and World Heritage is largely silent on how to overcome the ‘conflict of values’ that internationally significant areas often encompass. As noted above, both Ramsar and World Heritage have, over several decades, nursed core concepts intended to drive site management (and therefore impact assessment). In the case of World Heritage, this is the preservation of OUV, and in the case of Ramsar, it is the maintenance of ecological character. At the local scale, however, approaches to site management for these areas are unlikely to focus on OUV or ecological character unless specifically regulated. In practice, most World Heritage and Ramsar sites utilise national parks, marine parks, national monuments, State reserves or other locally dedicated arrangements as their basis for World Heritage or Ramsar site management. Very few States have World Heritage or Ramsar specific legislation that requires OUV or ecological character to be at the heart of management planning. In the absence of approaches that directly embed OUV or ecological character into local policy or legislative settings, practitioners may encounter discretion (or experience confusion) as to which frameworks to utilise, and, consequently, which specific values and threats to identify.

Discretion and confusion already abound in the practice of CEA, and the layering of additional international frameworks has the potential to further complicate decision-making, including with respect to cumulative impacts. To illustrate, it is well documented in the EIA literature that the practice of CEA involves the selection of ‘Valued Ecosystem Components’ (VECs) (Beanlands and Duinker 1983; Sinclair, Doelle, and Duinker 2017). In fact, as Duinker and Greig (2006) point out, CEA ‘demands’ a VEC-centred approach. The selection of VECs is highly subjective, and, in the literature, little is known about what influences decision-making about VECs (Olagunju and Gunn 2013). Indeed, it is possible that VEC selection may be determined by several factors, such as ecological significance, public value, or regulatory requirements (Ball, Noble, and Dubé 2013). The extent to which internationally embedded concepts (such as OUV or ecological character) influence VEC decision-making is further unknown. Questions may arise, for example, such as: can the Statement of OUV (in the case of World Heritage) or the Ecological Character Description (in the case of Ramsar) be used as a proxy for listing VECs in the undertaking of CEA? Or, might such an approach risk neglecting other, locally relevant, (but not less significant) components of the system?

Aligning the global and local scale in the context of environmental assessment is not an easy task. One of the challenges has traditionally been that decision-makers have lacked a systematic way of thinking about and addressing assessment and management across multiple scales of governance (Cash and Moser 2000). Some of this may be able to be overcome, for example, by utilising mediating institutions as a conduit between stakeholders as well as better coordinating the allocation of resources and technical expertise (Cash and Moser 2000). Whilst models of co-governance have some potential, there are often too many legal, policy and structural barriers impeding CEA collaboration (Margerum 2021). Because of the problem of ‘institutional fit’, large and complex socio-ecological systems (including many World Heritage and Ramsar sites) may require a ‘nested governance’ or hierarchical approach (Wyborn and Bixler 2013) although this comes with its own problems especially in the case of internationally significant areas where the values of local and Indigenous actors do not always align with those of ‘the State’.

### Incremental Adjustments Through Empirical Evaluation

Notwithstanding some of the deficiencies of the regimes (above) there are recent examples of addressing cumulative effects that may help to guide further policy development. Further empirical research of these cases may lend themselves towards important insights into how policy and guidance material may be further refined at the international scale. The case of Australia’s World Heritage listed Great Barrier Reef (GBR), for example, demonstrates the capacity of national and sub-national actors to think more critically about cumulative impacts affecting internationally significant areas. For the best part of two decades, the GBR has suffered from increasing intensity of land use impacts including from sugarcane, grazing, port development, over-fishing, tourism and climate change. In 2014, the Australian and Queensland governments responded to a call from UNESCO and the IUCN to conduct a comprehensive SEA for the GBR including strategically assessing policies, plans and procedures relevant to both terrestrial and marine components. One of the most positive outcomes from the entire exercise (which took two years to complete) was the development of the GBR’s first cumulative impact management policy (Hamman, Baresi and Vella 2021). The purpose of the policy is to provide ‘a systematic and consistent approach to managing and reducing cumulative impacts’ (Great Barrier Reef Marine Park Authority 2018). The SEA for the GBR undertook what appeared to be a CEA utilising, at least to an extent, the guidelines produced by the World Heritage Convention. No analysis, however, has been conducted of the extent to which the SEA complied with the guidelines or whether there were challenges with applying the prescribed material in practice.

In another example of CEA in a World Heritage area, Canada undertook an assessment of all existing and reasonably foreseeable developments impacting the World Heritage listed Wood Buffalo National Park (WBNP), including hydroelectric development; oil sands development; pulp and paper facilities; industrial mines; forestry activities; and municipal developments (UNESCO World Heritage Centre 2025a). Like the GBR, as requested by the World Heritage Committee, the exercise was one in SEA, not CEA, per se, although addressing cumulative effects was the main driver for requesting the assessment in the first place. There is evidence that the consultant engineers for the Canadian government did conduct a ‘high level’ CEA in order to gain an ‘understanding of the potential effects on the desired outcomes for the WBNP over the next thirty years’ (IEC 2018). The CEA for the WBNP reportedly utilised current stressors and trends already being observed in the Park as the baseline to which the potential cumulative effects of future development and climate change were factored in (IEC 2018). Again, however, no empirical analysis has been conducted of the extent to which Canada’s SEA complied with the World Heritage impact assessment guidelines.

As a final point, both the GBR and WBNP examples hint at a close, perhaps even ‘symbiotic’, relationship between SEA and CEA. Whilst they are not the same thing, the integration of CEA and SEA has long been attractive to policy makers because of the recognition that cumulative stresses are perceived to be best addressed at the strategic scale (Canadian Environmental Assessment Research Council 1986). On the face of it, both examples show that SEA can act as a governance prompt for closer consideration of cumulative effects. However, although it may appear seamless, the integration of CEA with SEA is often hampered by conceptual and methodological challenges (Gunn and Noble 2011). Questions still remain, therefore, about whether these are able to be overcome, including through enhanced guidance material. That said, even with enhanced guidelines for conducting CEA, alternative approaches may still need to be considered (Joseph et al., 2023), such as the extent to which Indigenous Peoples and local communities have been made central to the process (Adams et al. 2023; Kwon, Rutherford and Gunton 2024).

## Conclusion

Navigating the cumulative threats that impact upon the world’s most sensitive places continues to be a key challenge. International environmental law has a role to play in coordinating and legitimising structured approaches towards CEA, but hitherto, such measures have been haphazard and fragmented. In the absence of explicit acknowledgement in the text of the treaties themselves, regimes like World Heritage Convention and Ramsar have evolved, administratively, to develop guidance and other material that assists States in conducting EIA, including with a view to combatting cumulative effects. This article has traced the development of EIA and CEA under both the Ramsar and World Heritage regimes. It has examined the early work of the technical bodies under those Conventions (as well as the CBD). The evaluation of the current guidance material concluded that ambiguity and a weighted focus towards the ‘material’ or ‘biophysical’ (especially for Ramsar) may further undermine the practice of CEA in the field. Moreover, the question of competing value systems in the context of internationally protected areas may, in the long run, prove challenging in terms of scoping the most relevant threats as well as establishing a baseline and conducting a prospective analysis.

It is open to conclude that both Ramsar and the World Heritage Convention have adopted approaches towards CEA that are broadly consistent with the four steps used in the methodological approach. However, the comprehensiveness of the studied approaches remains subject to debate, especially when considered in the context of the wider academic literature and the differences between conducting CEA at the project level as opposed to CEA at the strategic scale. Whilst this paper does not fully explore solutions to these challenges, some practical steps include the need for incremental changes to current guidance (especially tightening the use of language), exploring alternative approaches to ‘traditional’ CEA (Joseph et al. 2023; Adams et al. 2023; Kwon, Rutherford and Gunton 2024) and publishing case studies of how CEA can or should be conducted in World Heritage and Ramsar listed locations. In this regard, further empirical research is warranted to better understand how practitioners and domestic governments might best approach the question of CEA in the context of internationally protected areas. The layering of international frameworks creates complexities for environmental management, but such challenges are not insurmountable. They are likely to require a high degree of regulatory cohesion and coordination. Key areas for academic inquiry could include examining the frameworks and guidelines that practitioners utilise to conduct their work in internationally significant places, as well as identifying the factors that influence VEC decision making where locations have been labelled with ecological character and/or OUV.

## Data Availability

No datasets were generated or analysed during the current study.

## Footnotes

1 International law also includes decisions of international courts and tribunals as well as customary international law, that is, where State practice indicates that a rule or norm has come into existence because it is generally accepted practice of States. There are no relevant (CEA) cases that the author is aware of in relation to the former. With respect to the latter, whilst there is some indication that environmental impact assessment (EIA) has reached the status of customary international law, any such discussion is outside the bounds of this article which focuses on different approaches towards CEA, specifically, and not the obligation to conduct EIA, generally.

2 This requirement was introduced in 2005. Sites on the List inscribed before 2005 require a Retrospective Statement of OUV which reflects the OUV at the time of listing of the site.

3 Whilst the use of definition(s) is indeed welcomed for the sake of clarity, it seems preoccupied with ‘project-level’ EIA, rather than CEA which should also occur for SEA (which focuses on policies, plans and programs, not ‘projects’).
